# 淋巴浆细胞淋巴瘤/华氏巨球蛋白血症诊断与治疗中国指南（2022年版）

**DOI:** 10.3760/cma.j.issn.0253-2727.2022.08.002

**Published:** 2022-08

**Authors:** 中国抗癌协会血液肿瘤专业委员会 中华医学会血液学分会 中国华氏巨球蛋白血症工作组

淋巴浆细胞淋巴瘤/华氏巨球蛋白血症（lymphoplasmacytic lymphoma/Waldenström macroglobulinemia，LPL/WM）是一种少见的惰性成熟B细胞淋巴瘤，在非霍奇金淋巴瘤中所占比例<2％。自《淋巴浆细胞淋巴瘤/华氏巨球蛋白血症诊断与治疗中国专家共识（2016年版）》[1]发布以来，我国医务工作者对该病的认识逐渐提高。近年来，LPL/WM发病机制、诊断和治疗均取得较大进展，为进一步促进我国LPL/WM规范化诊疗，提高我国LPL/WM患者疗效，经国内相关专家讨论，制定本指南。

一、定义

LPL是由小B淋巴细胞、浆样淋巴细胞和浆细胞组成的淋巴瘤，常常侵犯骨髓，也可侵犯淋巴结和脾脏，并且不符合其他可能伴浆细胞分化的小B细胞淋巴瘤诊断标准[2]–[3]。LPL侵犯骨髓同时伴有血清单克隆性IgM丙种球蛋白时诊断为WM。90％～95％的LPL为WM，仅小部分LPL患者分泌单克隆性IgA、IgG成分或不分泌单克隆性免疫球蛋白，诊断为非WM型LPL。由于非WM型LPL所占比例低，相关研究较少，治疗部分仅探讨WM的治疗，非WM型LPL的治疗参照WM进行。

二、诊断、分期、预后和鉴别诊断

（一）WM诊断标准[2]

1. 血清中检测到单克隆性IgM（不论数量）。

2. 骨髓中浆细胞样或浆细胞分化的小淋巴细胞呈小梁间隙侵犯（不论数量）。

3. 免疫表型：CD19（+），CD20（+），sIgM（+），CD5（−），CD10（−），CD22（+），CD23（−），CD25（+），CD27（+），FMC7（+），通常CD38和（或）CD138（+），而CD103（−）。但是，10％～20％的患者也可表达CD5、CD10或CD23。

4. 除外其他已知类型的淋巴瘤。

5. 90％以上WM发生MYD88 L265P突变[4]，但MYD88 L265P突变不是WM特异性突变，也可见于其他小B细胞淋巴瘤、弥漫大B细胞淋巴瘤等。

（二）鉴别诊断

1. 与IgM型意义未明的单克隆免疫球蛋白血症（MGUS）、多发性骨髓瘤（MM）等鉴别：

（1）IgM型MGUS[2]：IgM型MGUS的诊断标准：①有血清单克隆IgM蛋白；②骨髓中无淋巴浆/浆细胞浸润；③无其他B淋巴细胞增殖性疾病的证据；④无相关器官或组织受损的证据，如淋巴瘤浸润所致的贫血、肝脾肿大、高黏滞血症、系统性症状或淋巴结肿大，以及浆细胞疾病所致的溶骨性损害、高钙血症、肾功能损害或贫血。

（2）IgM相关性疾病[1]：这类患者存在由于单克隆性IgM升高引起的相关症状，如症状性冷球蛋白血症、淀粉样变，或自身免疫现象如周围神经病、冷凝集素病，但无淋巴瘤证据时，应诊断为IgM相关性疾病。

（3）IgM型MM：IgM型MM非常少见，细胞形态学为浆细胞形态，免疫表型为高表达CD38、CD138，而CD19、CD45阴性，常伴溶骨性损害等，这些特征是IgM型MM与WM鉴别的主要标志。MM常伴有14q32（IGH）易位，在WM中罕见，此外MM一般不伴MYD88基因突变，可作为两者的鉴别点。

2. 与其他B淋巴细胞增殖性疾病（B-LPD）鉴别：

多种B-LPD可伴有血清单克隆性IgM成分，并出现浆细胞分化的形态学特征，从而需与WM鉴别，如慢性淋巴细胞白血病/小淋巴细胞淋巴瘤、套细胞淋巴瘤、滤泡性淋巴瘤、边缘区淋巴瘤（MZL）、大B细胞淋巴瘤呈小细胞侵犯骨髓时，不典型的WM和MZL伴有浆细胞分化时尤其难以鉴别。

WM诊断及与其他B-LPD的鉴别涉及多个层面，而非某单一因素，怀疑WM时可遵循图1思路进行鉴别诊断，必要时还可参照《B细胞慢性淋巴增殖性疾病诊断与鉴别诊断中国专家共识（2018年版）》[5]。

**图1:**
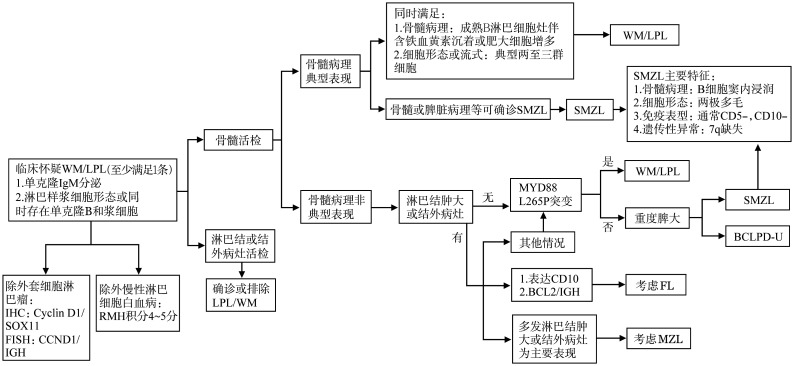
华氏巨球蛋白血症鉴别诊断流程图 WM/LPL：华氏巨球蛋白血症/淋巴浆细胞淋巴瘤；SMZL：脾边缘区淋巴瘤；MZL：边缘区淋巴瘤；FL：滤泡性淋巴瘤；BCLPD-U：不能分类的B细胞慢性淋巴增殖性疾病；重度脾大：脾下缘超过脐水平线，或脾右侧最远端超过腹中线，或彩超示脾脏下缘超过肋缘下6 cm

（三）分期和预后

包括两个预后分期系统：WM的国际预后指数（IPSSWM）和最新修订的国际WM预后积分系统（rIPSSWM），见表1、表2[6]–[8]。

**表1:** 华氏巨球蛋白血症国际预后评分系统（IPSSWM）[6]

积分		预后分层				
因素	分值	危险度	分值	比例（％）	中位总生存时间（月）	5年总生存率（％）
年龄>65岁	1	低危	0或1分且年龄≤65岁	27	142.5	87
HGB≤115 g/L	1	中危	2分或年龄>65岁	38	98.6	68
PLT≤100×10^9^/L	1	高危	>2分	35	43.5	36
β_2_-微球蛋白>3 mg/L	1					
血清IgM水平>70 g/L	1	

**表2:** 修订的国际华氏巨球蛋白血症（WM）预后积分系统（rIPSSWM）[7]

积分		预后分层					
因素	分值	危险度	分值	比例（％）	3年WM相关性死亡率（％）	5年总生存率（％）	10年总生存率（％）
年龄<65岁	0	极低危组	0	13	0	95	84
年龄66～75岁	1	低危组	1	33.5	10	86	59
年龄>75岁	2	中危组	2	25.5	14	78	37
β_2_-微球蛋白>4 mg/L	1	高危组	3	16	38	47	19
LDH>250 IU/L	1	极高危组	4～5	12	48	36	9
白蛋白<35 g/L	1						

MYD88突变阴性的WM通常预后更差，DNA损伤修复基因（TP53/ATM/TRRAP）突变[9]，特别是TP53缺失/突变是WM重要的不良预后因素[10]–[12]。

三、治疗

（一）治疗指征

无症状的WM患者不需要治疗。WM治疗指征为[13]：明显乏力、B症状、症状性高黏滞血症；WM相关的周围神经病变；淀粉样变；冷凝集素病；冷球蛋白血症；疾病相关的血细胞减少（HGB≤100 g/L、PLT<100×10^9^/L）；髓外病变，特别是中枢神经系统病变（Bing-Neel综合征）；症状性淋巴结肿大或器官肿大；有症状的肿大淋巴结或淋巴结最大直径≥5 cm；或有证据表明疾病转化时。单纯血清IgM水平升高不是本病的治疗指征。若血细胞减少考虑是自身免疫性因素所致，首选糖皮质激素治疗，若糖皮质激素治疗无效，则针对原发病治疗。

（二）治疗前评估

治疗前（包括复发患者治疗前）应对患者进行全面评估，应至少包括：

1. 病史（包括详细的既往史和家族史）和体格检查（特别是淋巴结和脾脏大小，有无周围神经病变表现）。

2. 体能状态评分：如美国东部肿瘤协作组体能状态评分（ECOG评分）。

3. B症状：发热、盗汗、体重减轻。

4. 血常规检查：包括白细胞计数及分类、血小板计数、血红蛋白、网织红细胞等。

5. 血生化检测：肝肾功能、电解质（血钙）、LDH、β_2_-微球蛋白等。

6. 免疫学检测：①免疫球蛋白定量：至少包括IgM、IgA、IgG水平；②血清蛋白电泳；③血免疫固定电泳；④24 h尿蛋白定量；⑤肝炎病毒检测。

7. 病理检查：①骨髓活检+涂片+免疫组化+流式细胞术分析；②淋巴结/其他组织病理+免疫组化（若可取）；③骨髓液或肿瘤组织进行MYD88 L265P突变检测，推荐使用液滴数字PCR（ddPCR）技术进行检测，敏感性可达0.01％[14]–[15]，或采用二代测序技术（NGS）检测，测序深度2 000×以上，敏感性可达1％，NGS至少包括MYD88和CXCR4，可同时检测ARID1A、TP53、TBL1XR1、ATM、TRRAP等。有条件的单位建议使用CD19磁珠分选后细胞进行检测，敏感性更高。

8. 影像学检查：颈、胸、腹部增强CT检查，怀疑有转化的患者建议做PET-CT。

其他检查包括：眼底检查；直接抗人球蛋白试验（怀疑有溶血时必做）、冷球蛋白和冷凝集素检测；神经功能相关检查（怀疑周围神经病时可查抗MAG抗体和抗GM1-4抗体）等。

（三）一线治疗选择[16]

对于有治疗指征的患者，首先推荐纳入设计良好的临床试验。无合适临床试验时，主要依据患者年龄、主要症状、合并疾病、治疗意愿、MYD88/CXCR4突变状况等选择治疗方案（详见表3，推荐流程见图2）。

**表3:** 华氏巨球蛋白血症治疗方案（按英文字母顺序排列，除非特别说明，均为2A类推荐）

优选方案	其他方案
①BR：苯达莫司汀+利妥昔单抗（R）[17] ②BDR：硼替佐米+地塞米松+R[18]–[19] ③伊布替尼单药或伊布替尼+R（1类推荐）[20]–[21] ④RCD：R+环磷酰胺+地塞米松[22] ⑤泽布替尼单药（1类推荐）[23]	苯达莫司汀 硼替佐米±R 硼替佐米+地塞米松 卡非佐米+R+地塞米松 克拉屈滨±R 苯丁酸氮芥±R 氟达拉滨±R FCR：氟达拉滨+环磷酰胺+R IRD：伊沙佐米+R+地塞米松 RCP：R+环磷酰胺+泼尼松 R单药

**图2:**
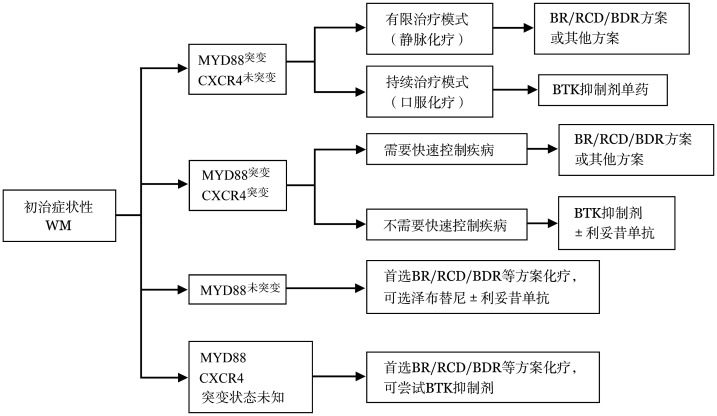
初治华氏巨球蛋白血症（WM）患者治疗推荐流程图

方案选择时注意以下几点：

1. 伴有症状性高黏滞血症的患者，建议先行血浆置换2～3次，后续以系统治疗。避免直接应用利妥昔单抗单药治疗，特别是IgM大于40 g/L时。

2. 主要症状为免疫相关的血细胞减少或器官肿大者，首选含利妥昔单抗为基础的方案化疗，如BR方案或RCD方案，可以较快降低肿瘤负荷。

3. 伴有IgM相关的神经性病变患者，应避免使用有潜在神经毒性的药物如硼替佐米等，建议使用含利妥昔单抗或BTK抑制剂为主的方案治疗。

4. 选择BTK抑制剂时需要结合MYD88 L265P/CXCR4突变状态：MYD88 L265P基因突变/CXCR4野生型患者疗效最好，推荐BTK抑制剂单药应用[24]；MYD88 L265P突变/CXCR4突变会降低BTK抑制剂疗效[23]，建议联合利妥昔单抗以提高疗效；MYD88野生型患者不推荐BTK抑制剂治疗，尤其是BTK抑制剂单药治疗。

5. 自体造血干细胞移植（ASCT）不作为一线治疗推荐。

（四）复发难治性患者的治疗选择

常规化疗复发患者仍然需要考虑是否具有治疗指征，无治疗指征的复发患者选择观察随访，有治疗指征的复发患者首选参加设计良好的临床试验。BTK抑制剂治疗后复发进展的患者，应持续应用BTK抑制剂至接受其他挽救治疗。其治疗方案选择同初治方案，主要选择和既往治疗非交叉耐药的方案。对于一线治疗3年后复发的患者，可继续应用原一线方案，而3年内复发的患者，应选择其他治疗方案[13]。BCL2抑制剂（Venetoclax）治疗复发难治患者的有效率为81％[25]，是BTK抑制剂治疗失败患者的重要选择。

ASCT是WM挽救治疗选择之一，对化疗仍敏感的复发患者，可考虑进行ASCT，特别是规范治疗后首次缓解时间小于2年或难治性患者，且BTK抑制剂充分治疗后进展或无效，推荐尽早进行ASCT（≤2次复发）[26]。发生转化的患者，应在大剂量化疗缓解后进行ASCT。因异基因造血干细胞移植较高的移植相关并发症发生率，仅在年轻、多次复发、原发难治/耐药，且一般状况较好的有合适供者的患者中选择性进行[26]。

（五）维持治疗

除非临床试验，不推荐常规进行维持治疗（注：回顾性研究表明利妥昔单抗联合治疗有效患者可从利妥昔单抗维持治疗中获益[27]，利妥昔单抗维持治疗：375 mg/m^2^，每3个月1次，连用2年。但对于BR方案治疗达到部分缓解以上的患者，利妥昔单抗维持治疗的生存获益不明显[28]）。

（六）中枢神经系统侵犯（Bing-Neel综合征）患者的治疗

中枢神经系统侵犯是WM一种罕见的并发症，中位发生时间为诊断WM后3～9年，表现多样，常见的症状包括四肢运动神经功能障碍、神志状态改变和颅神经麻痹，可侵犯脑实质或软脑膜。可选药物包括氟达拉滨、苯达莫司汀、大剂量甲氨蝶呤、阿糖胞苷等联合化疗，鞘内或脑室内注射甲氨蝶呤、阿糖胞苷和地塞米松也是一种有效治疗方式[29]–[30]。报道显示伊布替尼、泽布替尼均可有效治疗Bing-Neel综合征[31]–[32]。治疗有效者可考虑行ASCT巩固[33]。

（七）并发症的治疗

1. 贫血的治疗：贫血是本病最常见的临床表现和最主要的治疗指征，在疾病治疗起效前可应用rh-EPO、红细胞输注纠正或改善贫血。对于伴有高黏滞血症的患者，输注红细胞时应谨慎，以免增加血液黏滞度而加重患者症状；对于伴有冷凝集素综合征的患者，应输注预温至37 °C的红细胞；对于高血栓风险、高血压控制不良、肝功能不全的患者应慎用rh-EPO治疗。

2. 化疗相关性疱疹病毒感染：氟达拉滨、蛋白酶体抑制剂治疗过程中，约一半以上的患者可能出现疱疹病毒感染，应该进行疱疹病毒的预防性治疗，并持续至停药后6个月。

3. 利妥昔单抗治疗的燃瘤反应（flare现象）：利妥昔单抗单药治疗WM时可能出现燃瘤反应（发生率高达60％）[34]，即出现短暂的血IgM水平升高，加重高黏滞血症、冷球蛋白血症及其他IgM相关并发症。对于高IgM患者，特别是高于40～50 g/L的患者可考虑血浆置换，待IgM水平降低至约40 g/L以下后应用利妥昔单抗。但利妥昔单抗与其他药物联合，特别是与硼替佐米联合后燃瘤反应明显下降。

四、疗效标准

参照NCCN标准[16]，WM的疗效判断标准详见表4。需注意：①症状缓解是WM的首要目标，而不是缓解深度，目前治疗方案下大多数WM患者不能达到完全缓解，治疗有效的患者按原计划完成既定方案后停止治疗，不再为追求缓解深度而更换方案继续治疗。②WM起效相对缓慢，且通常临床症状如贫血的改善早于肿瘤负荷的降低，如无确切疾病进展证据，不宜频繁更换治疗方案。③由于血IgM定量受治疗的影响，如利妥昔单抗单药或联合化疗可能导致IgM水平升高并可能持续数月，而硼替佐米可能会较短时间内抑制IgM分泌但不杀伤肿瘤细胞，此时不能仅凭IgM定量来评价疗效，应该依据临床表现、血常规变化及影像学变化等进行综合评估，必要时进行骨髓活检等进行评判。

**表4:** 华氏巨球蛋白血症疗效评价标准[16]

疗效分组	判断标准
完全缓解（CR）	免疫固定电泳阴性并再次确认，IgM定量在正常范围；骨髓活检显示无骨髓受累；原有的髓外病灶消失，如肿大的淋巴结或脾脏；WM相关的临床症状及体征消失
非常好的部分缓解（VGPR）	血清IgM定量下降≥90%；原有的髓外病灶缩小，如肿大的淋巴结或脾脏；无新的疾病活动的症状或体征
部分缓解（PR）	血清IgM定量下降50%～90%；原有髓外病灶缩小，如肿大的淋巴结或脾脏；无新的疾病活动的症状或体征
微小反应（MR）	血清IgM定量下降≥25%但<50%；无新的疾病活动的症状或体征
疾病稳定（SD）	血清IgM定量增加或减少<25%；淋巴结肿大、脏器肿大、WM相关的贫血、临床症状体征无进展
疾病进展（PD）	血清IgM定量增加≥25%并需再次证实；或者由疾病本身导致的临床表现（如贫血、血小板减少、白细胞减少、淋巴结或脏器肿大等）或症状体征（如盗汗、不能解释的反复体温≥38.4 °C、体重减轻≥10%、高黏滞血症、神经病变、症状性冷球蛋白血症、淀粉样变性等）加重

五、随访

完成制定方案治疗或达疾病平台期的患者进入定期随访，前2年每3个月随访1次，随后3年每4～6个月随访1次，以后每年随访1次。随访内容包括病史、体格检查、血生化检查及IgM定量。影像学检查可依据患者具体情况决定，如前期有淋巴结/脏器肿大，可每3～6个月进行一次影像学复查，推荐增强CT检查。特别注意是否出现免疫性血细胞减少症（自身免疫性溶血性贫血、原发免疫性血小板减少症）、继发恶性肿瘤（包括弥漫大B细胞淋巴瘤、骨髓增生异常综合征、急性髓系白血病及实体瘤）等。

注：参照NCCN对证据和共识分类：1类：基于高水平证据，NCCN一致认为此项治疗合理；2A类：基于低水平证据，NCCN一致认为此项治疗合理；2B类：基于低水平证据，NCCN基本认为此项治疗合理；3类：基于任何水平证据，NCCN对此项治疗是否合理存在重大分歧。除非特殊说明，所有证据和共识均为2A类。
